# Methodological and ethical considerations in evaluating centralized COVID-19 drug procurement: a letter to the editor

**DOI:** 10.3205/id000095

**Published:** 2025-11-07

**Authors:** Muhammad Mustafa Khan, Abdullah Abdullah, Abdul Haseeb, Noor un nisa Irshad

**Affiliations:** 1 Khyber Medical College, Peshawar, Pakistan; 2 Jinnah Sindh Medical University, Karachi, Pakistan

## Letter to the editor

Dear editor,

We read with great interest the study by Marx et al. [1] titled “COVID-19 treatment strategies with drugs centrally procured by the German Federal Ministry of Health in a representative tertiary care hospital: a temporal analysis”, published in GMS Infectious Diseases. The authors provide valuable insights into centralized pharmaceutical procurement during the COVID-19 pandemic. However, we believe a broader discussion of methodological and ethical considerations is necessary to inform future public health planning and pharmaceutical policy fully.

The lack of a control group drastically reduces the capability of the study to infer causality. This limits the extent to which centralized procurement can be directly credited for observed outcome changes. Observed improvements in clinical outcomes, such as reduced mortality or shorter hospital stays, may result from changes in standard care procedures, enhanced clinician experience, or other temporal variables. To improve causal inference, this study could benefit from applying interrupted time series analysis to account for evolving pandemic dynamics, or propensity score matching to balance treatment groups and control for baseline confounding in the absence of randomization [2], [3].

In addition, the use of descriptive statistics only, without multivariable adjustment for important confounders such as comorbidities, age, or vaccination status, limits the study’s interpretability. The study did not attempt any statistical adjustments for potential confounders. A multivariable model, especially logistic regression (for binary outcomes like mortality) or Cox proportional hazards regression (for time-to-event outcomes), should have been used to control for confounding factors, providing more nuanced estimates of treatment effects [4]. Further, numerous reported standardized mean differences (SMDs<0.2) are below levels of clinical significance, questioning the value and cost-effectiveness of the interventions at emergency procurement.

The research only considers hospitalized patients, despite over 80% of COVID-19 patients being managed outside the hospital setting. This excludes the outpatient group population that accounts for a significant proportion of COVID-19 cases. This exclusion limits generalizability and overlooks the effects of early intervention measures, including oral antivirals such as nirmatrelvir/ritonavir, which have shown effectiveness in avoiding disease progression [5]. Subsequent analyses should include outpatient data to provide a more complete evaluation of public health interventions.

Use of unapproved or conditionally approved drugs under emergency procedures requires open ethical supervision. Individualized prescribing is referred to by the authors but not addressed in its implications. Without obvious regulatory channels and public accountability, this practice has the potential to undermine public trust, especially if side effects arise after the fact.

Furthermore, the research fails to critically evaluate the procedural and data-driven transparency, efficiency, or equity of centralized procurement versus decentralized approaches used in other nations. A more structured assessment factoring in procurement timelines, equity of access, and cost-benefit indicators is needed to inform future health system responses and avert misuse or corruption. In particular, such measures would enable comparison of outcomes within the study hospital with those of similar institutions that did not use the centralized procurement strategy [6]. 

The analysis offers data restricted to in-hospital outcomes, without any consideration for post-discharge trajectories such as long COVID, functional recovery, or readmission rates. This omission limits understanding of the full patient journey and may understate the real-world impact of treatment strategies. To provide a more comprehensive picture, it is crucial to incorporate validated post-discharge metrics. These include 30-day rehospitalization rates, emergency department re-visits, patient-reported outcome measures (PROMs), and functional assessments like the post-COVID-19 Functional Status (PCFS) scale or EQ-5D. Moreover, emerging literature suggests that post-acute sequelae of SARS-CoV-2 infection (PASC) significantly burden health systems and patients alike, often requiring long-term management and resources. Inclusion of these indicators would allow for more accurate evaluation of the sustainability and holistic effectiveness of centralized procurement strategies. It would also align the study with patient-centered care models that emphasize outcomes beyond discharge events, ultimately informing resource allocation, follow-up planning, and system resilience.

We applaud the authors for their work in a critical field of pandemic response, but we encourage subsequent studies to use a multidisciplinary and longitudinal design, blending causal inference, outpatient information, ethical considerations, and procurement transparency, to develop more actionable and equitable public health lessons. Only through such a holistic approach will preparedness be maximized and policy made with optimal effectiveness for future health crises.

## Notes

### Availability of data and material

The data that support the findings of this study are available from the corresponding author upon reasonable request.

### Authors’ contributions

All authors contributed to the conception, design, and writing of the manuscript. All authors read and approved the final manuscript.

### Author’s ORCID

Noor un nisa Irshad: 0009-0006-1584-5024

### Competing interests

The authors declare that they have no competing interests.

## References

[1] Marx K, Kalbitz S, Kellner N, Fedders M, Lübbert C (2023). COVID-19 treatment strategies with drugs centrally procured by the German Federal Ministry of Health in a representative tertiary care hospital: a temporal analysis. GMS Infect Dis.

[2] Pithon MM (2013). Importance of the control group in scientific research. Dental Press J Orthod.

[3] Kamper SJ (2018). Control Groups: Linking Evidence to Practice. J Orthop Sports Phys Ther.

[4] Riley RD, Jackson D, Salanti G, Burke DL, Price M, Kirkham J, White IR (2017). Multivariate and network meta-analysis of multiple outcomes and multiple treatments: rationale, concepts, and examples. BMJ.

[5] Petersen JJ, Jørgensen CK, Faltermeier P, Siddiqui F, Feinberg J, Nielsen EE, Torp Kristensen A, Juul S, Holgersson J, Nielsen N, Bentzer P, Thabane L, Kwasi Korang S, Klingenberg S, Gluud C, Jakobsen JC (2023). Drug interventions for prevention of COVID-19 progression to severe disease in outpatients: a systematic review with meta-analyses and trial sequential analyses (The LIVING Project). BMJ Open.

[6] Wang J, Zhang S, Wang C, Li J, Wang R, Zhu L (2024). The vacated space of volume/price of the drugs centralized procurement with quantity in secondary and above public hospitals of China. BMC Health Serv Res.

